# Filtering and Imaging of Frequency-Degenerate Spin Waves Using Nanopositioning of a Single-Spin Sensor

**DOI:** 10.1021/acs.nanolett.2c02791

**Published:** 2022-10-21

**Authors:** Brecht G. Simon, Samer Kurdi, Joris J. Carmiggelt, Michael Borst, Allard J. Katan, Toeno van der Sar

**Affiliations:** † Department of Quantum Nanoscience, Kavli Institute of Nanoscience, Delft University of Technology, 2628 CJDelft, The Netherlands

**Keywords:** Spin-wave imaging, scanning nitrogen-vacancy magnetometry, diamond, yttrium iron garnet, quantum sensing

## Abstract

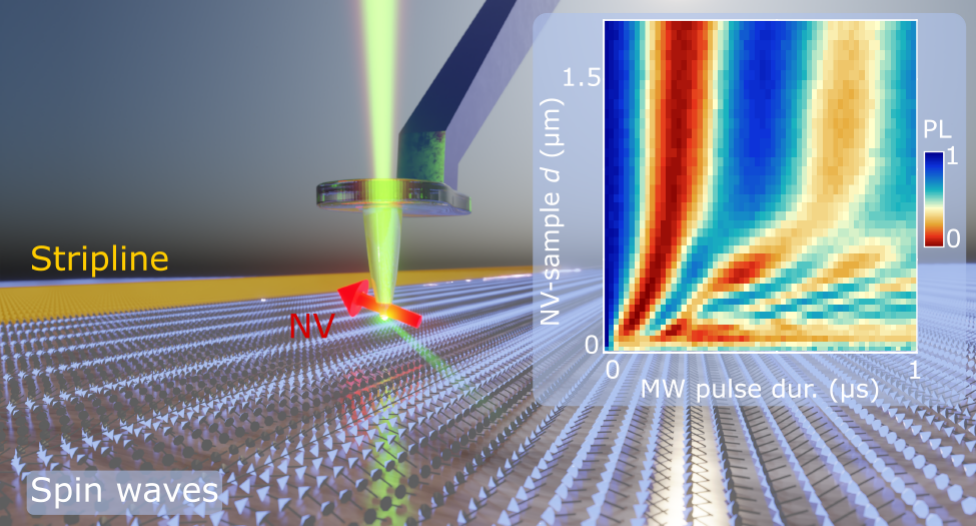

Nitrogen-vacancy (NV) magnetometry is a new technique for imaging spin waves in magnetic materials. It detects spin waves by their microwave magnetic stray fields, which decay evanescently on the scale of the spin-wavelength. Here, we use nanoscale control of a single-NV sensor as a wavelength filter to characterize frequency-degenerate spin waves excited by a microstrip in a thin-film magnetic insulator. With the NV probe in contact with the magnet, we observe an incoherent mixture of thermal and microwave-driven spin waves. By retracting the tip, we progressively suppress the small-wavelength modes until a single coherent mode emerges from the mixture. In-contact scans at low drive power surprisingly show occupation of the entire isofrequency contour of the two-dimensional spin-wave dispersion despite our one-dimensional microstrip geometry. Our distance-tunable filter sheds light on the spin-wave band occupation under microwave excitation and opens opportunities for imaging magnon condensates and other coherent spin-wave modes.

Spin waves are collective spin excitations of magnetically ordered materials, with associated quasi-particles called magnons.^1^ Due to their low damping, spin waves are promising as information carriers in information-technology devices.^2−5^ Techniques to image spin waves aid in studying such devices and realizing their technological potential. As such, several imaging techniques have been developed, with most established techniques based on the spin-dependent scattering of photons.^6−8^

Nitrogen-vacancy (NV) magnetometry images spin waves by their microwave magnetic stray fields. It uses the electronic spin of the NV lattice defect in diamond as a sensor, which can be read out through spin-dependent photoluminescence (PL), is atomic-sized, and can stably exist within nanometers from the diamond surface.^9,10^ This enables magnetic imaging with nanoscale spatial resolution and high sensitivity. The NV spin allows probing spin-wave spectra with a ∼1-MHz frequency resolution through spin lifetime measurements and characterizing spin-wave amplitudes by measuring the NV spin rotation rate.^11^ Recently, NV magnetometry has been used to study domain-wall-guided spin-wave modes,^12^ magnon scattering,^13−15^ spin chemical potentials,^16^ and frequency combs.^17^ To enable sensitivity to target spin-wavelengths, accurate control of the NV-sample distance is crucial because the spin-wave stray fields depend exponentially on the distance to the sample at a length scale set by their wavelength.

Here, we demonstrate that controlling the NV-sample distance using a diamond tip mounted on an atomic force microscope (Figure 1a) creates a tunable wavelength filter that enables selective probing of frequency-degenerate spin-wave modes. Increasing the NV-sample distance progressively filters out small-wavelength spin waves, enabling studies of long-wavelength modes that are otherwise hidden in thermal spin-wave noise. We demonstrate high-contrast imaging over a range of wavelengths by adjusting the NV-sample distance on the nanoscale. When maximizing the wavenumber-cutoff of our distance-tunable filter via in-contact scans, we find a surprising pattern of standing spin waves instead of the expected traveling waves. Fourier transforms of the patterns reveal an occupation of spin-wave modes along the entire isofrequency contour of the two-dimensional spin-wave dispersion despite our one-dimensional stripline geometry, which we attribute to spin-wave scattering. Within this ensemble, we clearly identify spin waves with wavelengths of only 360 ± 20 nm. These results show that the exponential decay of the spin-wave stray fields provide a resource unique to magnetic-resonance spin-wave imaging, enabling wavenumber-selective detection of frequency-degenerate spin waves and high-resolution imaging of spin-wave scattering.

**Figure 1:**
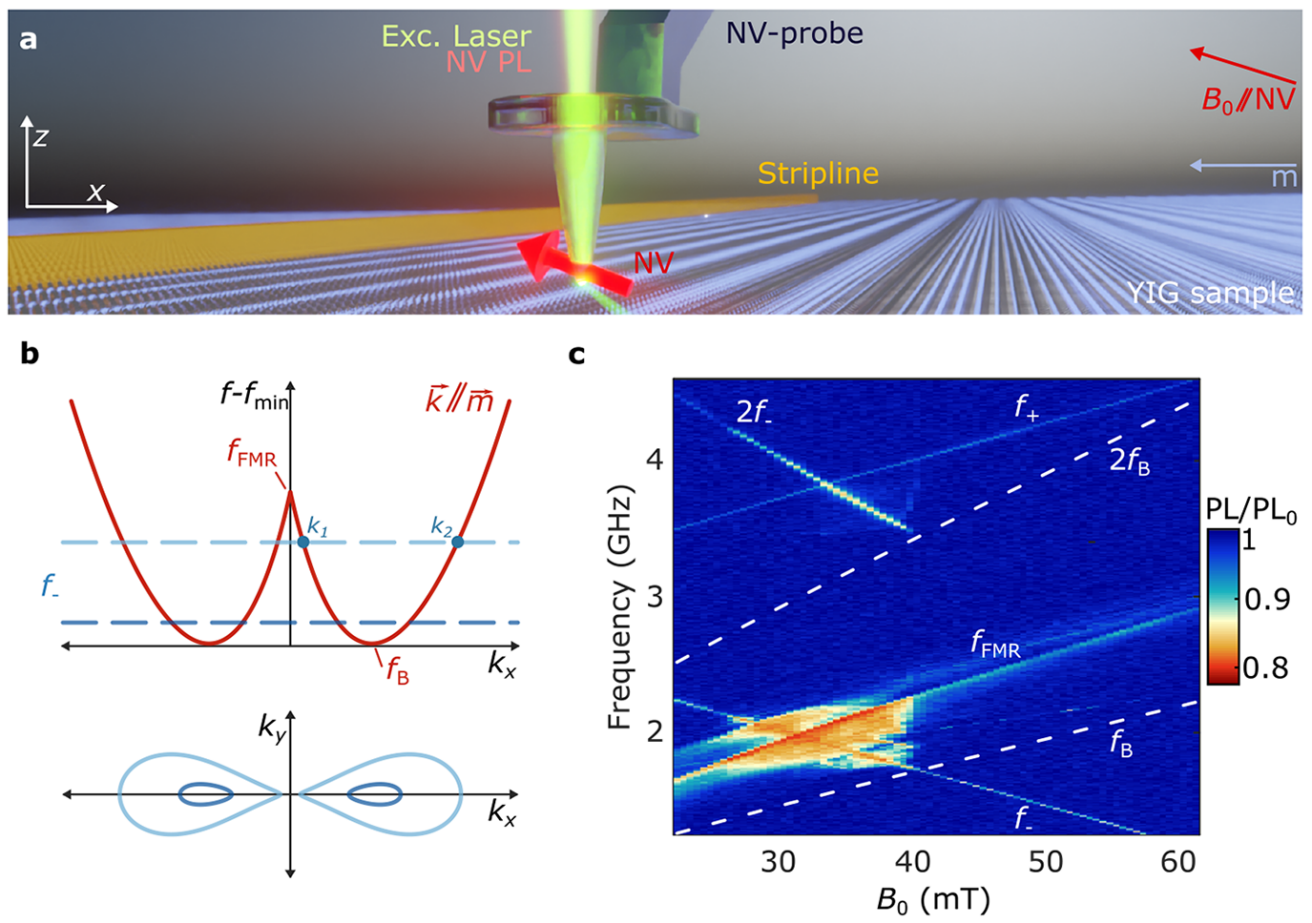
Imaging stripline-driven spin waves using scanning nitrogen-vacancy (NV) magnetometry. (a) A single NV spin embedded ∼20 nm from the apex of a diamond tip (Supporting Information, Note 2) measures the magnetic stray fields of spin waves excited by a microwave stripline in a 235-nm-thick yttrium iron garnet (YIG) film. The NV spin is initialized using a green laser and read out via its spin-dependent photoluminescence (PL). A bias magnetic field *B*_0_ is applied along the NV axis, magnetizing the film perpendicularly to the 1-mm-long, 15-μm-wide stripline. (b) Top: calculated dispersion of spin waves traveling parallel to the YIG magnetization (“backward-volume” spin waves). The NV spin detects spin waves at its electron spin resonance (ESR) frequency, *f*_–_, indicated by dashed lines for two values of *B*_0_ (darker color corresponds to a larger *B*_0_). In this work, we focus on spin waves resonant with *f*_–_ in the range *f*_B_ < *f*_–_ < *f*_FMR_ for which there exist two frequency-degenerate backward volume modes, *k*_1_ and *k*_2_. Bottom: isofrequency contours of the two-dimensional spin-wave dispersion at the frequencies indicated by the dashed lines in the top panel. (c) NV photoluminescence as a function of *B*_0_ and the microwave drive frequency. Data taken with the NV tip in contact with the YIG at ∼30 μm from the stripline edge at 1 mW drive power. The NV photoluminescence under microwave excitation (PL) is normalized to the NV photoluminescence without microwave excitation (PL_0_). The ESR frequencies (*f*_±_) and calculated FMR frequency *f*_FMR_ are labeled. The dashed lines indicate the calculated minimum spin-wave frequency *f*_B_ and its harmonic at 2*f*_B_.

Our system consists of a thin film of yttrium iron garnet (YIG), a magnetic insulator with ultralow spin-wave damping.^18^ We excite spin waves by applying a microwave current to a stripline that is microfabricated onto the YIG surface (Figure 1a, Supporting Information, Notes 1–3). We apply a bias field *B*_0_ along the NV axis to tune the NV electron spin resonance (ESR) frequencies (*f*_±_) relative to the spin-wave band. The orientation of *B*_0_ magnetizes the film in-plane and perpendicularly to the stripline, enabling efficient excitation of “backward-volume” spin waves^18^ that travel parallel to the magnetization (Figure 1b, Supporting Information, Notes 4 and 5). The spin waves generate magnetic stray fields above the surface that drive our NV spin when resonant with an NV ESR frequency. We detect these NV-resonant spin waves via the NV center’s spin-dependent photoluminescence^19^ (PL).

We start by providing an overview of the NV PL as a function of the frequency of the microwave current applied to the stripline and the bias field *B*_0_ (Figure 1c). We do so with the diamond tip in contact with the YIG at ∼30 μm from the stripline. We observe several regions of reduced PL caused by NV spin transitions that provide a first insight into the spin waves excited by the stripline: first, two lines of reduced PL occur when the drive frequency is resonant with the NV ESR frequencies *f*_–_ and *f*_+_. Here, the NV spin is driven by the sum of the direct stripline field and the stray field of spin waves excited by the stripline.^11,14^ Second, a line of reduced PL reveals the YIG ferromagnetic resonance (FMR). Here, FMR-induced magnon–magnon scattering leads to spin-wave noise at the NV frequencies that causes NV spin relaxation and an associated PL reduction.^13,16^ Third, we observe a broad region of reduced PL when *f*_–_ is in the vicinity of the FMR. In this region, the stripline efficiently excites spin waves because of their micron-scale wavelengths near the FMR (Supporting Information, Note 5). These spin waves in turn scatter efficiently to modes resonant with *f*_–_ because they are close in frequency and wavelength,^20^ causing NV spin relaxation. Correspondingly, the region of reduced PL ends abruptly when *f*_–_ drops below the bottom of the spin-wave band (labeled *f*_B_ in Figure 1c) at *B*_0_ ≈ 41 mT. In this work, we study spin waves in the region *f*_B_ < *f*_–_ < *f*_FMR_ and use the nanoscale control of the NV tip as a wavelength filter to separate the contributions from frequency-degenerate incoherent and coherent spin waves.

Spin waves generate a rotating magnetic stray field with amplitude *B*_SW_ that decays with increasing distance *d* to the sample,^21^ with the decay length set by the spin-wavenumber *k* according to
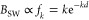
1where *f*_*k*_ is the filter function (Figure 2a,b). As such, increasing the NV-sample distance progressively filters out the stray fields of high-wavenumber spin waves. We demonstrate the filtering by characterizing the stray fields of spin waves excited by the microwave stripline as a function of the NV-sample distance. We do so by measuring the NV spin rotation rate (Rabi frequency), which depends linearly on the amplitude of the NV-resonant microwave field. We measure the Rabi frequency by tuning the NV frequency *f*_–_ to the isofrequency contour of Figure 1b and applying variable-duration microwave pulses (Figure 2c). These pulses excite *f*_–_-resonant spin waves that drive NV spin rotations via their magnetic stray field.^22^

**Figure 2:**
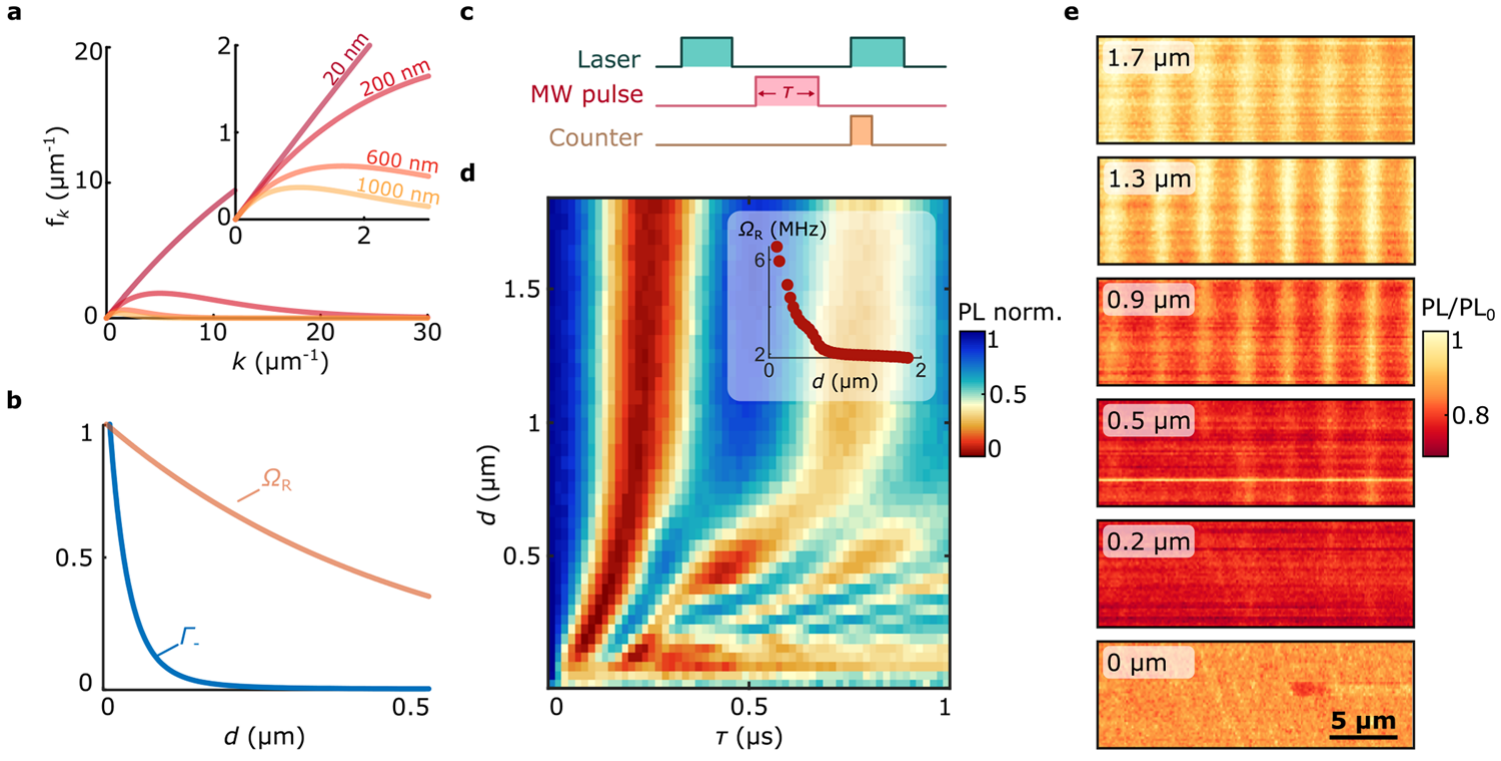
Tuning the NV-sample distance as a filter to selectively image a long-wavelength spin-wave mode. (a) The spin-wave stray field is proportional to a prefactor *f*_*k*_ (filter function) that depends on the NV-sample distance *d* (eq 1). The filter function peaks at *k* = 1/*d*, where *k* is the spin-wavenumber. The filter function is plotted for several values of *d*. (b) Calculation comparing the NV relaxation rate Γ_–_ caused by thermal spin waves to the NV Rabi oscillation rate caused by a coherently driven spin wave. Both rates are normalized to their value at *d* = 0 to highlight the different scaling with distance. The calculation of Γ_–_ assumes an equal population of all spin-wave modes at frequency *f*_–_ as expected for a Rayleigh-Jeans distribution.^21^ The calculation of Ω_R_ assumes only a single spin-wave mode with wavenumber *k* = 2 μm^–1^ (as imaged in e) is excited. (c) Pulse sequence used for the measurement in d: A 2.5 μs green laser pulse initializes the NV spin. A variable-duration microwave (MW) pulse excites spin waves. The final NV spin state is read out by measuring the NV photoluminescence during the first 600 ns of a second green laser pulse. (d) Spin-wave-driven NV spin dynamics vs tip–sample distance *d*. The dynamics are governed by the stray-field spectrum of the spin waves at the NV frequency. For *d* ≳ 100 nm, the stray field of a coherent spin wave yields high-visibility Rabi oscillations with a long decay time. Below ∼100 nm, the Rabi decay time starts to vanish, attributed to the more rapidly increasing stray field generated by thermal spin waves (see b). Measurement taken at 31 μm from the stripline edge at *B*_0_ = 32 mT, *f*_–_ = 1.98 GHz, and *P*_MW_ = 6.3 mW. Inset: Fitted Rabi frequency vs *d* down to 100 nm. (e) Spatial maps of the ESR contrast while driving spin waves at *f*_–_ = 1.89 GHz and *B*_0_ = 35 mT (*k*_1_ = 1.7 μm^–1^ and *k*_2_ = 22 μm^–1^) at *P*_MW_ = 1 mW for varying *d*. The ESR contrast is obtained by normalizing the NV photoluminescence under microwave excitation (PL) to that without microwave excitation (PL_0_).

With the tip in contact with the YIG (Figure 2d, *d* = 0 nm), we observe fast NV spin decoherence, indicating a strong presence of incoherent spin-wave noise. As further shown below, the noise is caused by a combination of thermal and microwave-excited spin wave modes. By lifting the NV a few hundred nanometers, we suppress the noise sufficiently and start observing NV Rabi oscillations, indicating a coherent microwave field at the NV frequency. The nonexponential decrease of the Rabi frequency with a further increasing *d* (Figure 2d and its inset) shows that the Rabi oscillations are driven by an ensemble of coherent spin waves of which the high wavenumbers are progressively suppressed by the distance-dependent cutoff of the filter. The microwave magnetic field generated by the stripline, which is approximately constant over the ∼2 μm lift-height range given the ∼30 μm distance to the stripline edge, sets the Rabi frequency at large *d*.

Using spatial maps of the ESR contrast (Figure 2e), we demonstrate that the distance-tunable filter enables spatial imaging of a single low-*k* spin wave within an ensemble of frequency-degenerate spin-wave modes. We define the contrast *C* by the ratio of the NV PL with and without microwave drive (*C* = 1 – PL/PL_0_). The spatial contrast arises due to the interference between the field of the excited spin waves (which are propagating) and the uniform reference field that is supplied by our stripline.^11,14^ The in-contact scan (bottom panel, Figure 2e) shows two important features: first, the maximum contrast, *C*_max_(*d* = 0) = 0.15, is reduced with respect to the maximum contrast at increased distances *C*_max_(*d* > 200 nm) = 0.25. Second, the contrast equals its maximum value throughout the scan (i.e., it is saturated). The reduced ESR contrast of the in-contact scan is consistent with the strong increase of the stray field generated by thermally excited spin waves (Figure 2b) that enhance the NV-spin relaxation rate^16,21,23−25^ (Supporting Information, Note 6) and lead to PL reduction.^26,27^ The spatially homogeneous saturation indicates a large amplitude of the microwave-driven spin waves, as we will show in more detail below.

Retracting the tip to *d* = 0.2 μm, we find that the contrast approximately doubles with respect to *d* = 0 μm. This is expected from the rapid suppression of the thermal spin-wave stray fields by our filter. However, we still find that the contrast is saturated over the entire spatial map (Figure 2e) due to the large stray fields of spin waves excited by the microwave drive. For distances *d* > 1 μm, the microwave-driven spin waves are filtered to an extent that yields a clear spatial image of a single low-*k* spin-wave mode (Figure 2e). These results show how lifting the tip from the surface filters out high-*k* spin waves, enabling high-contrast imaging of a single low-*k* spin wave within an ensemble of thermal and coherent spin-wave modes.

Fast NV-imaging of spin waves requires a strong ESR contrast. Because the spin-wave stray field falls off exponentially (eq 1), maintaining a strong contrast requires adapting the NV-sample distance to the expected spin-wavelength (Figure 3a). We change the wavelength of the mode, indicated by *k*_1_ in Figure 1b, by increasing *B*_0_ while reducing the drive frequency according to *f*_–_ = *D* – γ*B*_0_ to maintain resonance with the NV, where *D* = 2.87 GHz is the NV zero-field splitting and γ = 28 GHz/T is the electron gyromagnetic ratio. Starting from the distance used in Figure 2c for *B*_0_ = 35 mT, we find that keeping *kd* constant yields high-contrast images over a range of wavelengths (Figure 3b). The spatial images of Figure 3b show how the wavelength decreases with increasing *B*_0_ until the *f*_–_ detection frequency drops below the bottom of the spin-wave band at *B*_0_ ≈ 41 mT (inset Figure 3c). At this field (*B*_0_ = 41 mT), both modes (labeled *k*_1_ and *k*_2_ in Figure 1) are expected to contribute to the interference pattern. However, due to the low signal-to-noise we cannot conclusively identify both modes (Supporting Information, Note 7). The large ESR contrast enables a straightforward extraction of the wavelengths (Supporting Information Note, 7), which correspond well with the calculated spin-wave dispersion (Figure 3c).

**Figure 3:**
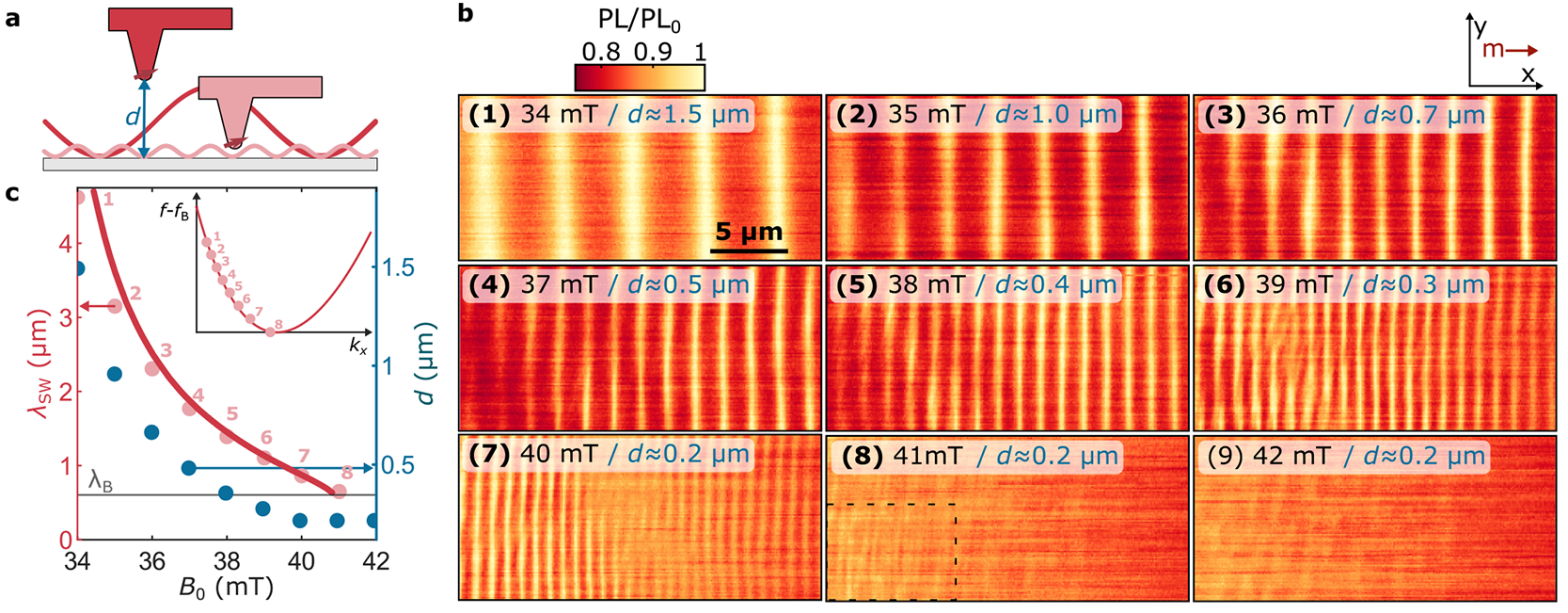
Adapting the sensor-to-sample distance to realize high-contrast imaging of different spin-wavelengths. (a) Spin waves generate a magnetic stray field that decays exponentially at the scale of the spin-wavelength. We tune the tip–sample distance *d* to optimize the detection of different wavelengths. (b) Spatial maps of the NV ESR contrast showing backward-volume spin waves excited by the stripline at different magnetic fields *B*_0_. Increasing *B*_0_ (panels 1–8) decreases the wavelength of the spin waves that are resonant with the NV ESR frequency. In each scan, we tune the distance *d* to maintain a constant ESR contrast, as plotted in c. In panel 8, we extract the wavelength by analyzing the dashed box. Drive power *P*_MW_ = 4 mW. (c) The wavelengths extracted by fitting (Supporting Information, Note 7) the wave patterns in b (pink dots) compared to the wavelengths calculated from the backward-volume dispersion (plotted in red, Supporting Information, Note 4), as a function of *B*_0_. The wavelength at the minimum of the spin-wave band is indicated by λ_B_. Green dots (right *y*-axis): the tip–sample distance *d* used in each of the scans shown in b. Inset, red line: calculated dispersion of the backward-volume spin waves relative to the minimum spin-wave frequency. Pink dots: modes imaged in b.

Bringing the NV-tip into contact with the sample maximizes the wavenumber cutoff of our filter and increases the relative contribution of high-wavenumber modes to the stray field (Figure 2a). We use in-contact scans to study the ensemble of spin-wave modes excited in the magnetic film. To avoid the spatially homogeneous saturation of the NV ESR contrast observed for the in-contact scan of Figure 2e, we reduce the microwave drive power by a factor of 500. This reduction yields smaller amplitudes of the microwave-excited spin waves, which prevents saturation of the NV ESR contrast and thereby re-establishes the ability of the ESR contrast to be modulated by spatial changes in the spin-wave stray fields. The resulting spatial modulations of the ESR contrast reveal a rich pattern of spin waves in different directions (Figure 4a).

**Figure 4:**
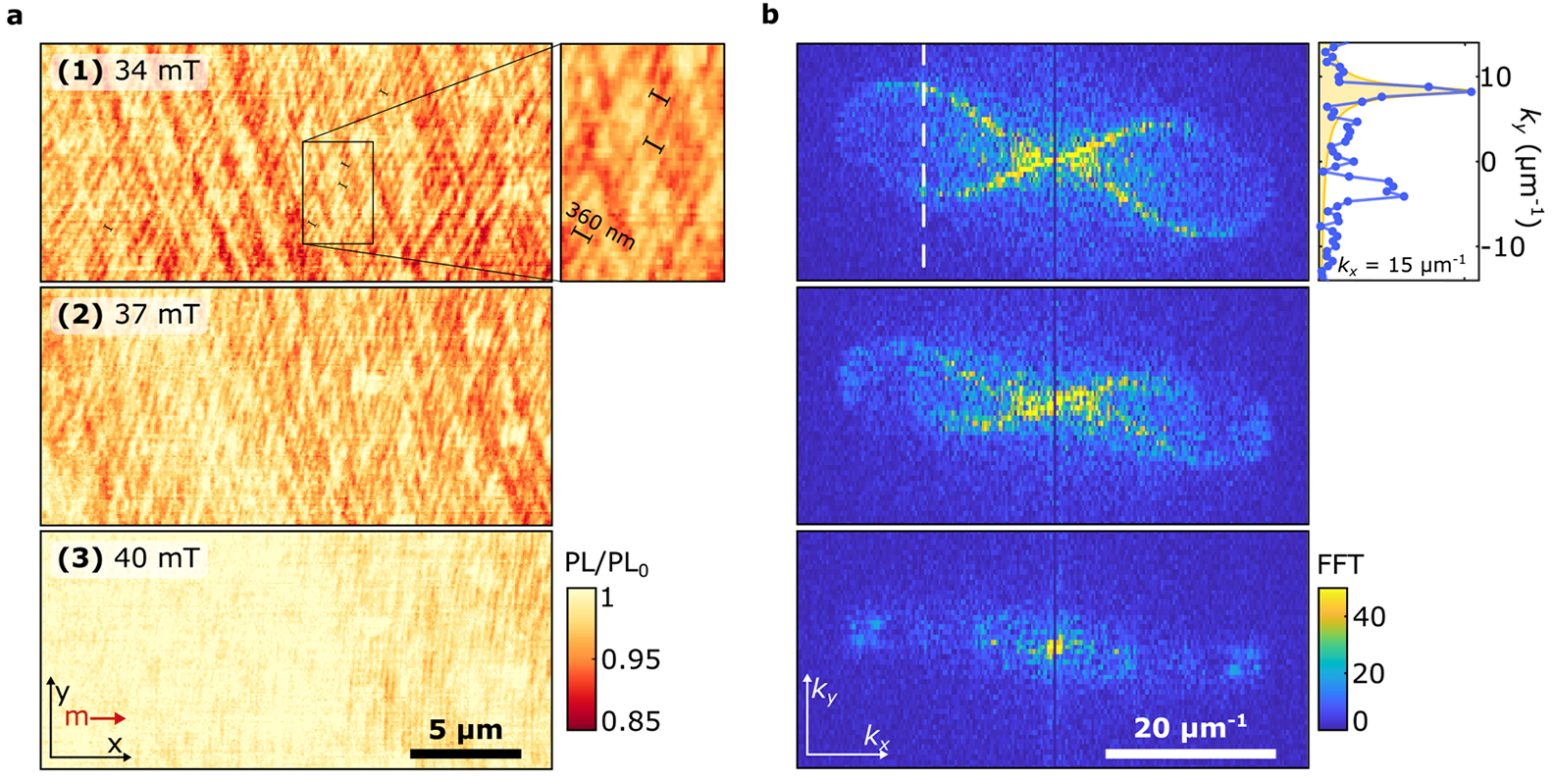
Emergence of frequency-degenerate standing spin-wave modes in spatial maps of the ESR contrast. (a) Measured ESR contrast when the tip is in contact with the sample at low drive power (6.3 μW) for different magnetic bias fields *B*_0_. Right panel: oom-in of 1 with 360 nm spin waves. (b) Absolute value of the Fourier transformations (FFT) of the maps in a, revealing the wavevectors present in the spatial spin-wave patterns. The full isofrequency contour of the spin-wave dispersion is visible at *B*_0_ = 34 mT. Right panel: linetrace at *k*_*x*_*=* 15 μm^–1^ (white dashed line). The fitted peak (yellow) corresponds to *k*_*y*_ = 8.3 μm^–1^, yielding *k* = 17 μm^–1^ and θ_*k*_*=* 29°. The corresponding wavelength of 360 ± 20 nm (uncertainty derived from the fitted peak width) is clearly visible in the zoomed-in real-space image presented in 1 (black bars).

To interpret the wavenumber content of the spin-wave patterns observed in Figure 4a, we perform a Fourier transform. The Fourier maps reveal the excitation of spin-wave modes along the entire *f*_–_-isofrequency contour of the two-dimensional spin-wave dispersion (Figure 4b). We observe spectral content at wavenumbers up to *k* ≈ 25 μm^–1^. The peaks at *k* ≈ 17 μm^–1^ (right panel, Figure 4b) correspond to spin-wavelengths of ∼360 nm that are also clearly visible in the real-space images (right panel, Figure 4a). Although these modes are not directly excited by our microstrip, such an homogeneous occupation of the spin-wave dispersion may be expected when taking into account scattering of the primarily excited backward volume spin waves^28^ enhanced by the presence of defects, such as small scratches and small pits that are homogeneously present in our liquid-phase-epitaxy-grown YIG film (Supporting Information, Note 8). Previous works^29−34^ demonstrated that such defects act as spin-wave scatterers and lead to the occupation of high-*k* modes.

The absence of ESR contrast for *B*_0_ > 40 mT (at which the NV frequency sits below *f*_B_; Supporting Information, Note 9) shows that the amplitude of the direct stripline field is too small to generate ESR contrast. We therefore conclude that the stray-field patterns observed at *B*_0_ < 40 mT do not result from interference between the spin-wave and stripline fields but are instead generated by standing spin waves that result from scattering. These results highlight the coherent nature of the scattering process and the efficiency by which it leads to the occupation of high-momentum modes that are otherwise inaccessible to a one-dimensional excitation stripline.

Nanoscale control of the NV-sample distance serves as a tunable filter that enables balancing the magnetic fields generated by an ensemble of incoherent and coherently driven spin waves of different wavelengths. This control enables selective imaging of a coherent spin-wave mode within a mixture of frequency-degenerate spin waves and retaining a high-visibility response when imaging different wavelengths. In-contact scans at reduced drive power show a surprising pattern of standing spin-wave modes. The Fourier transforms of these patterns reveal spin-wave occupation along the entire isofrequency contour of the two-dimensional spin-wave dispersion. We attribute the occupation of these high-momentum modes to defect-enhanced spin-wave scattering. The phase relation between the scattered modes is maintained, emphasizing the coherent nature of the scattering process. Nanoscale control of the NV-sample distance and wavenumber-selective imaging of magnetic oscillations at microwave frequencies paves the way for imaging magnon condensates^35^ or other coherent spin-wave modes^12^ and could also be used to probe microwave electric current distributions in devices.

## Notes

All data contained in the figures will be made available at zenodo.org upon publication with the identifier 10.5281/zenodo.6703953. Additional data related to this paper may be requested from the authors.

## Supporting Information Available

Details on the YIG sample, the measurement setup, and the spin-wave measurement methods; description of the calculation of the spin-wave dispersion, the stripline field, and the NV relaxation rate induced by thermal magnons; additional data on fitting the spin-wavelength, YIG surface topography and photoluminescence scans, and an overview of the spin-wave images taken at various fields and scan-modes; details on the calibration of the piezoelectric scanners (PDF)
