# Supplementary material for: Filtering and Imaging of Frequency-Degenerate Spin Waves Using Nanopositioning of a Single-Spin Sensor
Source: Nano Lett. 2022 Oct 21;22(22):9198–204. doi: 10.1021/acs.nanolett.2c02791 (PMC9706654; doi:10.1021/acs.nanolett.2c02791)
Supplement: Supplementary file 1 — nl2c02791_si_001.pdf [file nl2c02791_si_001.pdf]

# Supporting Information: Filtering and imaging of frequency-degenerate spin waves using nanopositioning of a single-spin sensor

Brecht G. Simon†, Samer Kurdi†, Joris J. Carmiggelt, Michael Borst, Allard J. Katan, Toeno van der Sar*

**Affiliations**
Department of Quantum Nanoscience, Kavli Institute of Nanoscience, Delft University of Technology, 2628 CJ, Delft, The Netherlands

† These authors contributed equally to this work.
* Corresponding author. Email: t.vandersar@tudelft.nl

S.1 YIG Sample . . . 2
S.2 Measurement Setup . . . 2
S.3 Spin-wave measurement methods . . . 2
S.4 Spin-wave dispersion . . . 2
S.4.1 Magnetic susceptibility . . . 2
S.4.2 Equilibrium magnetization . . . 3
S.4.3 Spin-wave dispersion . . . 3
S.5 Stripline field . . . 4
S.6 NV relaxation induced by thermal magnons . . . 4
S.7 Spatial ESR contrast generated by a single spin wave . . . 5
S.7.1 Extracting the spin wavelength of the low wavenumber mode . . . 6
S.8 Combined atomic force microscopy and photoluminescence scans of the YIG surface . . . 7
S.9 Overview spin-wave images . . . 8
S.10 Calibration of the piezoelectric scanners . . . 9
S.10.1 Lateral displacement . . . 9
S.10.2 Calibration of the NV-to-sample distance . . . 9

## S.1 YIG Sample

The $\sim 235(10)$ nm thick yttrium iron garnet (YIG) was grown on a gadolinium gallium garnet (GGG) substrate by liquid-phase epitaxy (Matesy GmbH). The YIG chip was first sonicated in acetone to remove contaminants. A 1 mm-long and 15 µm-wide stripline (5 nm titanium / 200 nm gold) for spin-wave excitation was then deposited on top of the YIG surface using $e$-beam evaporation preceded by $e$-beam lithography, using a double PMMA resist (A8 495K / A3 950K) and a top layer of Elektra92.

## S.2 Measurement Setup

Our scanning NV-magnetometry setup consists of two stacks of Attocube positioners (ANPx51/RES/LT) and scanners (ANSxy50/LT and ANSz50/LT) that enable individual positioning of the tip and sample, in addition to a confocal microscope setup, which are all placed in an acoustical enclosure. The confocal setup uses a 515 nm green laser (Cobolt 06-MLD, pigtailed) for NV excitation, which is focused by the objective lens (LT-APO/VISIR/0.82) onto a single-NV tip ((001)-oriented, QZabre Ltd). The NV was created via nitrogen implantation with an implantation energy of 7 keV. This energy leads to an expected implantation depth of about $(10 \pm 10)$ nm (see, e.g. Ref. [1]). Taking into account a possible selection of relatively deep NVs because of photoluminescence properties, we conservatively estimate an NV-depth of 20 nm below the tip surface. The NV photoluminescence (PL) is collected by the same objective and separated from the excitation laser by a dichroic mirror (Semrock Di03-R532-t3-25x36) and a long-pass filter (Semrock BLP01-594R-25), spatially filtered by a pinhole (50 µm), and finally collected by an avalanche photodiode (APD) (Excelitas SPCM-AQRH-13). A SynthHD (v2) microwave generator (Windfreak Technologies, LLC) was used to apply microwave signals. A programmable pulse generator (SpinCore Technologies, Inc. PulseBlasterESR-PRO 500) controls the timing of the laser excitation, detection window and microwaves. A National Instruments card (PCIe 6323) was used for the data acquisition.

## S.3 Spin-wave measurement methods

All measurements were performed close to the middle of the 1 mm-long stripline to prevent edge effects from stripline corners, within 30 µm from the edge of the 15 µm-wide stripline. The direct stripline field interferes with the spin-wave field to form the standing-wave stray-field patterns of figures 2 and 3 (main text) [2, 3]. The static field $B_0$ is applied by moving a small permanent magnet mounted on translation stages. For all measurements, the magnet is aligned along the NV axis within $\sim 5°$ such that the expected angle between the NV and the sample is $\theta \approx 54°$ with respect to the sample-plane normal. Because of some uncertainty introduced when mounting the NV-probe, we leave this angle as a free parameter when fitting the measured wavelength to the spin-wave dispersion, yielding $\theta = 49°$ (Fig. 3d). For the scans at non-zero tip-sample distances in figures 2 and 3 (main text), we first touch down onto the sample with the tip to acquire a well-defined distance reference. Then, we turn off the AFM feedback and set the lift height using our piezo scanner (Supporting Information, Note S.10). We repeat this for each line trace. As there is no feedback, the lift height can change over a line trace due to drift or sample tilt.

## S.4 Spin-wave dispersion

Here, we calculate the spin-wave dispersion of our 235 nm film of yttrium iron garnet (YIG). We assume a 2D geometry, where the magnetization does not change across the film thickness (Fig. S.1). We first consider the relevant energy contributions for our magnetic system to evaluate the Landau-Liftshitz-Gilbert (LLG) equation that describes the dynamics of the magnetization. Following the approach described by Rustagi *et. al*[4], we then obtain the magnetic susceptibility (section S.4.1) and the spin-wave dispersion (section S.4.3).

### S.4.1 Magnetic susceptibility

Given that the Zeeman interaction, the demagnetizing field and the exchange interaction are the relevant energy contributions, we calculate the response of the transverse magnetization $\delta\vec{m}'_\perp$ to a drive field $\vec{h}_\perp(\vec{k})$ via $\delta\vec{m}'_\perp = S\vec{h}_\perp$. Here, $\delta\vec{m}'$ is defined in the magnet frame, where the equilibrium magnetization ($\vec{m}_{\rm eq}$) points in the $z$-direction. And $S$ is the transverse magnetic susceptibility, which is given by [4]:

$$S(\vec{k},\omega) = \frac{\gamma}{\Lambda}\begin{bmatrix}\omega_3 - i\alpha\omega & -\omega_1 - i\omega \\ -\omega_1 + i\omega & \omega_2 - i\alpha\omega\end{bmatrix} \tag{S.1}$$

where

$$\omega_0(\vec{k}) = \omega_{\rm B}\cos(\theta_B - \theta_0) - \omega_{\rm M}\cos^2\theta_0 + \omega_D k^2, \tag{S.2}$$

$$\omega_1(\vec{k}) = \omega_{\rm M} f_L \sin\phi_k \cos\phi_k \cos\theta_0, \tag{S.3}$$

$$\omega_2(\vec{k}) = \omega_0 + \omega_{\rm M}\left[f_L\cos^2\phi_k\cos^2\theta_0 + (1-f_L)\sin^2\theta_0\right], \tag{S.4}$$

$$\omega_3(\vec{k}) = \omega_0 + \omega_{\rm M} f_L \sin^2\phi_k, \tag{S.5}$$

$$\Lambda(\omega) = (\omega_2 - i\alpha\omega)(\omega_3 - i\alpha\omega) - \omega_1^2 - \omega^2. \tag{S.6}$$

Here, $\omega_B = \gamma B_0$, is the frequency associated with the Zeeman energy, where $\gamma$ is the gyromagnetic ratio and $B_0$ the externally applied magnetic field. We apply $B_0$ at an angle $\theta_B$, which is the direction of the magnetic field with respect to the sample normal, such that it aligns with the NV center. As a result, the equilibrium magnetization $\theta_0$ tilts out-of-plane by an angle $\theta_0$. Next, the frequency associated with the demagnetizing field is given by: $\omega_M = \gamma\mu_0 M_s$, where $\mu_0$ and $M_s$ are the vacuum permeability and the saturation magnetization respectively. Finally, $\omega_D = \frac{\gamma D}{M_s}$ is associated with the exchange interaction, where $D$ is the spin stiffness[i]. A wave vector, $\vec{k}$ is described by its wavenumber $k$ (i.e. the modulus of the wave vector) and by its direction which is described by $\phi_k$. Finally, the prefactor $f_L$ is given by $f_L \equiv 1 - (1 - e^{-kL})/(kL)$ in which $L$ is the film thickness.

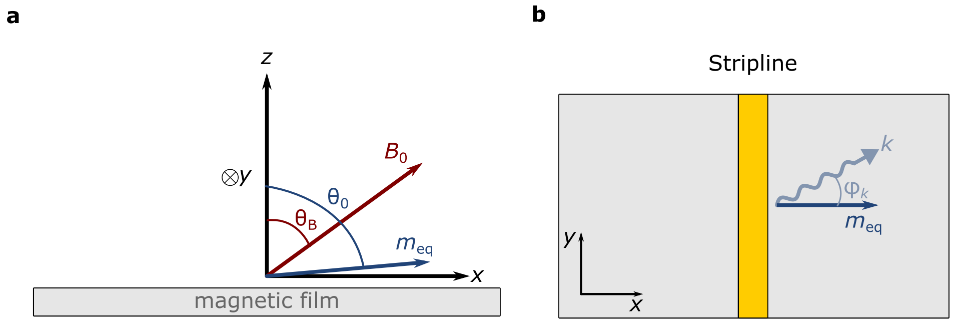


**Figure S.1: Schematic of the measurement geometry.** (a) Side view of our measurement geometry. The magnetic field is applied at an angle $\theta_B$ with respect to the sample normal. As a result the equilibrium magnetization $\vec{m}_{\rm eq}$ tilts slightly out-of-plane with an angle $\theta_0$. (b) Top view of the measurement geometry. We drive the transverse magnetization via an oscillating magnetic field supplied by a microwave current that is sent through a microstrip with width 15 µm and length 1 mm. In this work, the stripline field excites spin waves that travel parallel to the equilibrium magnetization, also called backward-volume spin waves.The parameters used for calculating the spin-wave dispersion for the film studied in this work are: $M_s = 1.42\cdot 10^5$ A/m, $A_{\rm ex} = 3.8\cdot 10^{-12}$ J/m, $\alpha = 1\cdot 10^{-4}$ and $L = 235$ nm [2]. The angle between the magnetic field and the film, $\theta_B$ is $\theta_B \approx \theta_{\rm NV} \approx 54°$.

### S.4.2 Equilibrium magnetization

The equilibrium angle of the magnetization, $\theta_0$, follows from minimizing the free energy and solving for each value of the magnetic field:

$$-2B_0\sin(\theta_B - \theta_0) = \mu_0 M_s \sin(2\theta_0) \tag{S.7}$$

We calculate that $\theta_0$ is in-plane to within a few degrees for the magnetic fields used in our measurements.

### S.4.3 Spin-wave dispersion

The spin-wave dispersion is given by the frequencies for which the susceptibility is singular, i.e. when: $\Lambda = 0$ (Fig. S.2).By tuning the external magnetic field, we vary $f_-$ with respect to the minimum spin-wave frequency, as such the contour (dashed line in Fig. S.2) changes shape such that $f_-$ becomes resonant with spin waves of different wavevectors.

[i]The spin stiffness is often expressed in terms of the exchange constant $A$ with more conventional units (J/m): $D = 2\gamma A_{\rm ex}/M_s$ (with units (rad/s/m$^2$))

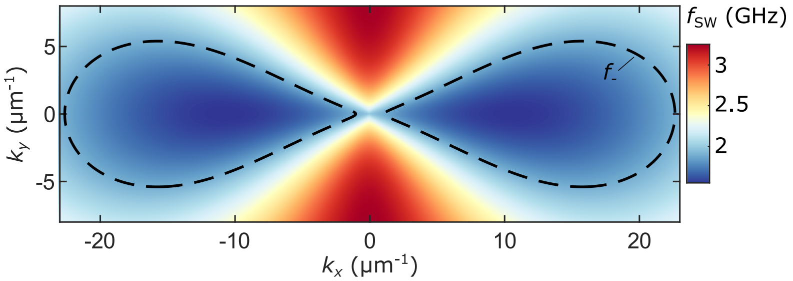


**Figure S.2: Spin-wave dispersion.** Calculated spin-wave dispersion for $B_0 = 35\,\text{mT}$, i.e. when $f_\text{B} < f_- < f_\text{FMR}$. The field is aligned along the NV-axis such that $f_- = 1.89$ GHz, which is represented by the iso-frequency contour (dashed line).

## S.5 Stripline field

We use a stripline oriented along $y$, with width $w$, length $L$ and thickness $h$ for spin-wave excitation, centered at $x = 0$ and $z = -h/2$. A microwave current density $J(\omega)$ applied to the stripline generates a magnetic field with components [2]:

$$h_\text{x} = 2J(\omega)e^{kz}\frac{e^{kh}-1}{kk_x}\sin(k_x\frac{w}{2})\frac{\sin(k_y\frac{L}{2})}{k_{y(x)}} \tag{S.8}$$

$$h_\text{z} = -2iJ(\omega)e^{kz}\frac{e^{kh}-1}{k^2}\sin(k_x\frac{w}{2})\frac{\sin(k_y\frac{L}{2})}{k_y} \tag{S.9}$$

$$\tag{S.10}$$

in k-space. Because the film is magnetized along $x$, the x-component of the field does not contribute to spin-wave excitation. As such, we only consider the $z-$component. The field exciting the spin waves is obtained by averaging over the film thickness:

$$\tilde{h}_z = -2iJ(\omega)\frac{e^{-kL}-1}{kL}\frac{e^{kh}-1}{k^2}\sin(k_x\frac{w}{2})\frac{\sin(k_y\frac{L}{2})}{k_y} \tag{S.11}$$

Because the length of the stripline far exceeds its width and the distance between the stripline center and our measurement location, it is essentially a one-dimensional stripline that does not excite spin waves in the $k_y$ direction (Fig. S.3).

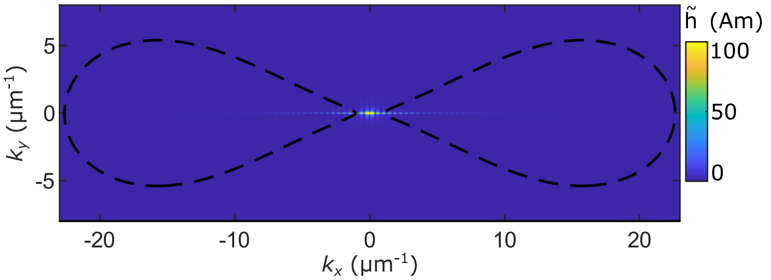


**Figure S.3: The effective field strength for a stripline that is aligned perpendicular to the magnetization (backward-volume geometry).** Using a 15 μm-wide and 1 mm-long stripline. The stripline field is most efficient in driving low-wavenumber modes (close to the FMR). Due to its one-dimensional character, spin waves with a component in the $k_y$ direction are not excited. Dashed line indicates the $f_-$-isofrequency contour (Fig. S.2) of NV resonant modes at 35 mT.

## S.6 NV relaxation induced by thermal magnons

We follow the approach of Rustagi *et al.*[4] to calculate the NV relaxation rates induced by the magnons in our YIG film (Fig. 2d, main text) using:

$$\Gamma_\mp(\omega_\mp) = \frac{\gamma^2}{2}\int\frac{d\vec{k}}{(2\pi)^2}\sum_{i,j\in\{x,y\}}\mathcal{D}^\text{eff}_{\pm i}(\vec{k})\mathcal{D}^\text{eff}_{\mp j}(-\vec{k})C_{ij}(\vec{k},\omega_\mp). \tag{S.12}$$

Here, $\Gamma_{\mp}$ are the relaxation rates corresponding to the $\omega_{\mp}$ ESR frequencies, $\vec{k}$ is the spin-wavevector, $\mathcal{C}$ is a spin-spin correlator describing the thermal magnon fluctuations, and $\mathcal{D}^{\text{eff}}$ is a dipolar tensor that calculates the magnetic stray fields that induce NV spin relaxation generated by these fluctuations. Note, this equation is defined in the magnet frame, for which the equilibrium magnetization is along the $z$-direction.

The thermal transverse spin fluctuations in the film are described by [4]:

$$C_{ij}(\vec{k},\omega) = 2D_{th} \sum_{\nu=\{x,y\}} S_{i\nu}(\vec{k},\omega) S_{j\nu}(-\vec{k},-\omega) \tag{S.13}$$

where $D_{\text{th}} = \frac{\alpha k_B T}{\gamma M_s L}$, with $k_B$ the Boltzmann constant, $T$ the temperature, $S$ the magnetic susceptibility (Eq. S.1).

The dipolar tensor $D^{\text{eff}}(\vec{k},\omega)$ is obtained by first rotating the magnet frame to the lab frame, then multiplying by the dipolar tensor $\mathcal{D}(\vec{k})$ in the lab frame, and then rotating the result to the NV frame: $D^{\text{eff}}(\vec{k},\omega) = R_{yz}(\theta_{\text{NV}},\phi_{\text{NV}})\mathcal{D}(\vec{k})R_Y(\theta_0)^T$, where

$$\mathcal{D}(\vec{k}) = -\frac{\mu_0 M_s}{2} e^{-|\vec{k}|d_{\text{NV}}}(1-e^{-|\vec{k}|L}) \begin{bmatrix} \cos^2\phi_k & \sin(2\phi_k)/2 & i\cos\phi_k \\ \sin(2\phi_k)/2 & \sin^2\phi_k & i\sin\phi_k \\ i\cos\phi_k & i\sin\phi_k & -1 \end{bmatrix}, \tag{S.14}$$

where $\mu_0$ is the vacuum permeability and $d_{\text{NV}}$ is the distance between the NV and the sample surface. The terms in Eq. (S.12) that induce spin relaxation are given by[4]: $\mathcal{D}^{\text{eff}}_{\pm\nu} = \mathcal{D}^{\text{eff}}_{x\nu} \pm i\mathcal{D}^{\text{eff}}_{y\nu}$.

For a magnon gas in thermal equilibrium, in the absence of microwave driving, the dependence of the NV relaxation rate on the NV-sample distance can be calculated using Eq. (S.12). The fast increase in rate (Fig. 2d, main text) results in a reduction of the ESR contrast as the NV sensor approaches the film to within nanometer proximity.

## S.7 Spatial ESR contrast generated by a single spin wave

Here, we determine the spatial profile of the ESR contrast generated by a propagating spin-wave mode, with wavenumber $k$ that interferes with an uniform reference field of varying amplitude (Fig. S.4)[2, 3]. We show that the ESR contrast spatially varies depending on the wavenumber given that the reference field has a finite amplitude.

Spin waves produce a field of which the component ($B_{\text{SW}}$) that is rotating with the correct handedness in a plane that is perpendicular to the NV-axis drives Rabi oscillations[2]. This component varies spatially according to:

$$B_{\text{SW}} = B^0_{\text{SW}} e^{ik(x-x_0)} \tag{S.15}$$

This field induces NV Rabi oscillations of which the rate is given by:

$$\Omega_R = \frac{\gamma}{\sqrt{2}}|B_{\text{SW}} + B_{\text{ref}}| \tag{S.16}$$

where $B_{\text{ref}}$ is the component of the reference field that is rotating with the correct handedness in a plane perpendicular to the NV-axis. The ESR contrast is given by:

$$\text{PL}/\text{PL}_0 = 1 - C_{\text{ESR}} \tag{S.17}$$

$$\text{PL}/\text{PL}_0 = 1 - \beta\frac{\Omega_R^2}{\Omega_R^2 + \delta^2} \tag{S.18}$$

where $\beta$ is a constant that describes the maximum ESR contrast and $\delta$ is a parameter that depends on the optical pumping rate[5], which we assume to be constant as all measurements were taken at the same laser power.

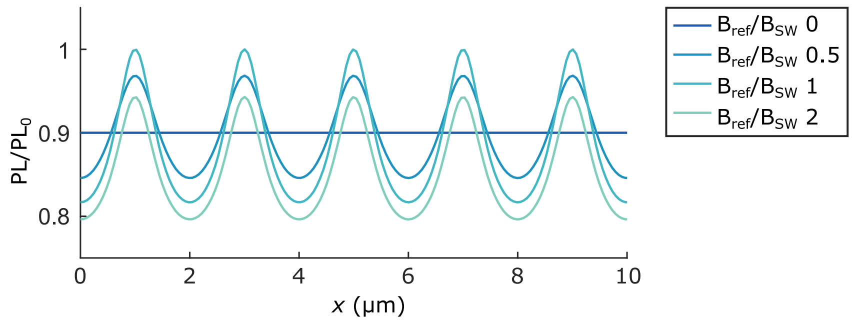


**Figure S.4: Spatial profile of a single spin wave mode interfering with a reference field.** The expected PL/PL$_0$ for a single spin-wave mode for various strengths of the reference field.

### S.7.1 Extracting the spin wavelength of the low wavenumber mode

Here we analyze the spatial maps shown in figure 3b (main text) and extract the wavelength of the imaged modes. To do so, we first plot the signal as a function of $x$ (Fig. S.5a), after subtracting a linear term to account for the non-uniformity of the stripline-field. Using equation S.18, we then fit the averaged data (Fig. S.5a). The fit allows to extract the wavenumber $k$, which we plot as a function of $B_0$ (Fig. 3c, main text). In figure S.5b, we show the corresponding Fourier transform of the averaged data traces.

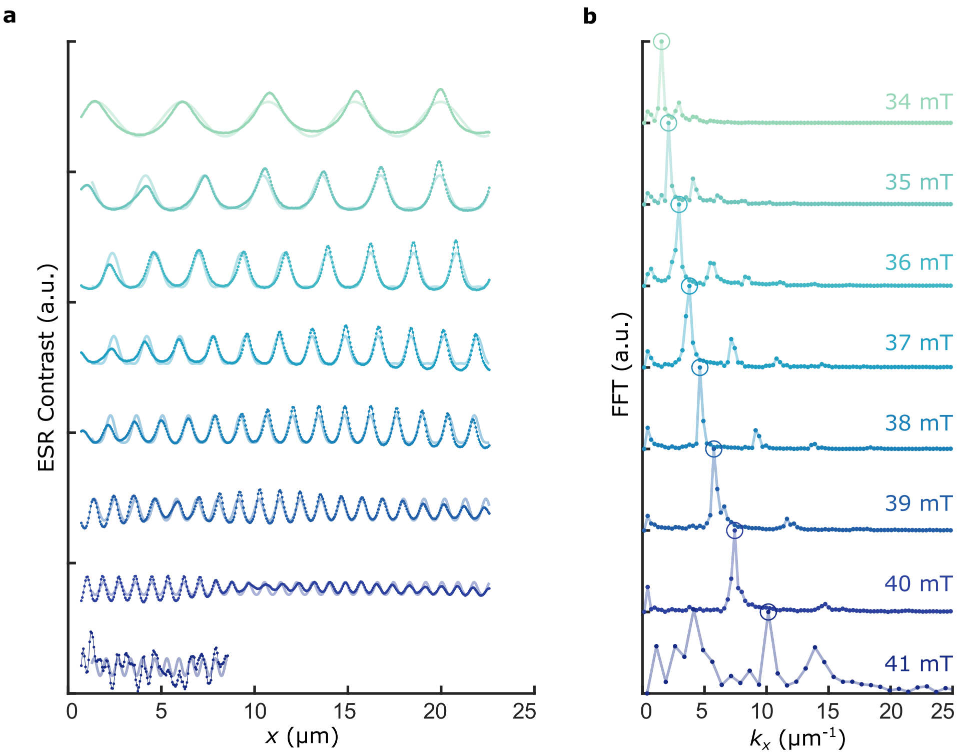


**Figure S.5: Wavelength analysis of spin-wave maps** (a) We average the 2D maps of figure 3b (main text), to obtain a 1D linetrace of the ESR contrast as a function of $x$-position. We fit the data using equation S.18, to obtain the wavenumber and its uncertainty. (b) Fourier transform of the 1D data shown in (a). Circles represent peak positions used as initial guess for the fitting.

Finally, we fit the extracted wavenumbers to the backward-volume spin-wave dispersion and we find that an angle of $\theta_B = 49°$, which is the angle between the magnetic field and the YIG surface normal, fits our data best, due to an uncertainty in mounting of the NV-probe with respect to the sample surface.

## S.8 Combined atomic force microscopy and photoluminescence scans of the YIG surface

In contact mode, our scanning NV magnetometry setup collects both the topography, via the AFM feedback signal, and the NV photoluminescence (Fig. S.6). The topography image not only shows dirt particles laying on top of the surface (large white dot in the center of the scan), but also scratches and tiny pits in the YIG surface that can lead to magnon scattering [6–8].

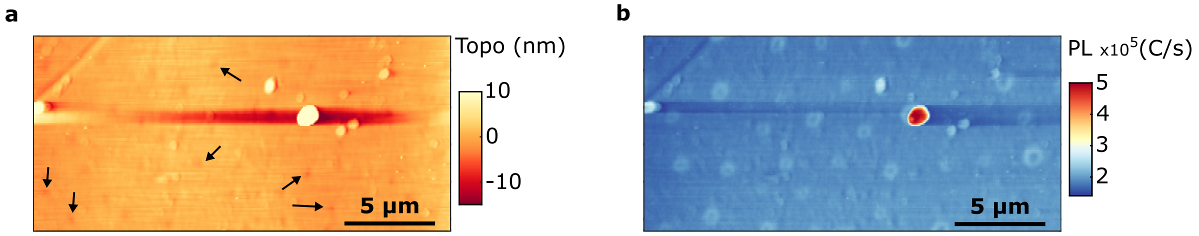


**Figure S.6: YIG surface.** (a) Surface topography (Topo) where the arrows indicate several small defects and (b) photoluminescence (PL) of our 235 nm-thick YIG film surface. Data corresponds to the ESR map in figure S.7b, scan 1 taken with the tip in contact with the YIG surface.

## S.9 Overview spin-wave images

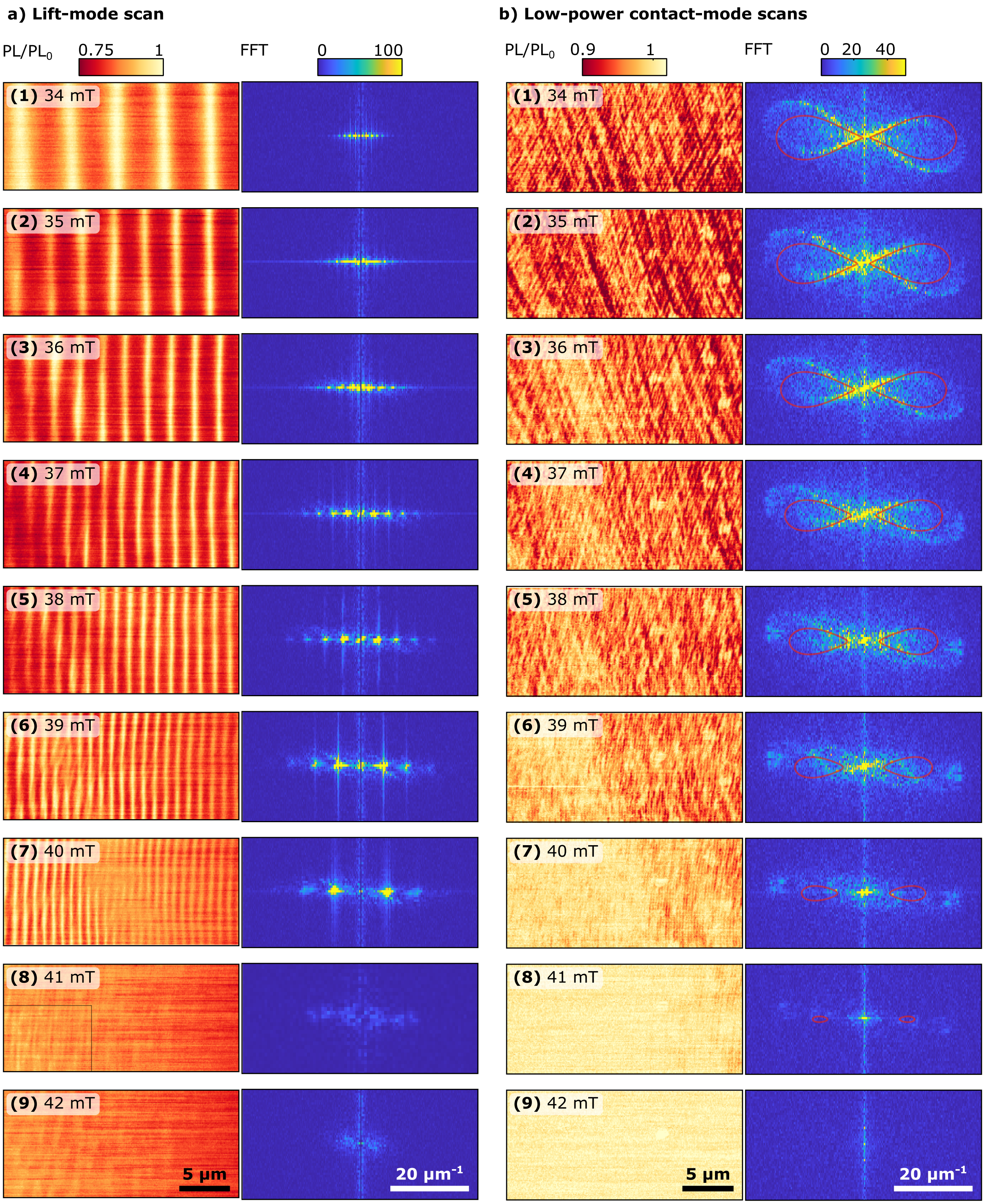


**Figure S.7: Overview spin-wave images** (a) Scans corresponding to Fig. 3b of the main text using $P_{\mathrm{MW}} = 4\,\mathrm{mW}$ and we change the NV-to-sample depending on the spin-wavelength. (b) Scans corresponding to Fig. 4a of the main text using $P_{\mathrm{MW}} = 6.3\,\mu\mathrm{W}$ while keeping the NV-tip in contact with the magnetic surface. Red line: the calculated isofrequency contour of the 2D spin-wave dispersion.

## S.10 Calibration of the piezoelectric scanners

### S.10.1 Lateral displacement

Our piezoelectric scanner (ANSxy50/LT) exhibits a nonlinear motion as a function of applied voltage. We calibrate this non-linear motion using a silicon nitride sample with 2 µm chess pitch structures. Specifically, we scan over the same region of interest and piezoscanner voltages/offsets while recording the photoluminescence (Figure S.8a). Our calibration procedure to convert the non-linear displacement as a function of applied voltage to position is as follows:

1. We first remove the first 25 lines from our scan data which show large non-linear and non-reproducible displacements depending on scan-speed and time spent on the first pixel.
2. We assign the applied voltage to known positions of subsequent chess pitches (Fig. S.8). For simplicity, we do this for a single row or column (Fig. S.8b).
3. We fit a second order polynomial to the known X or Y displacement.

We repeat this process for the two scan speeds used in this work: $0.1\,\mathrm{V\,s^{-1}}$ and $0.05\,\mathrm{V\,s^{-1}}$ used for the lift-mode and contact mode scans. We can now interpolate our 2D spatial scans, that were taken at linearly spaced voltage interval and obtain a 2D image with linearly spaced position intervals. Note that all scan data shown in the manuscript are taken in the forward scan direction and we therefore do not take piezoelectric hysteresis into account.

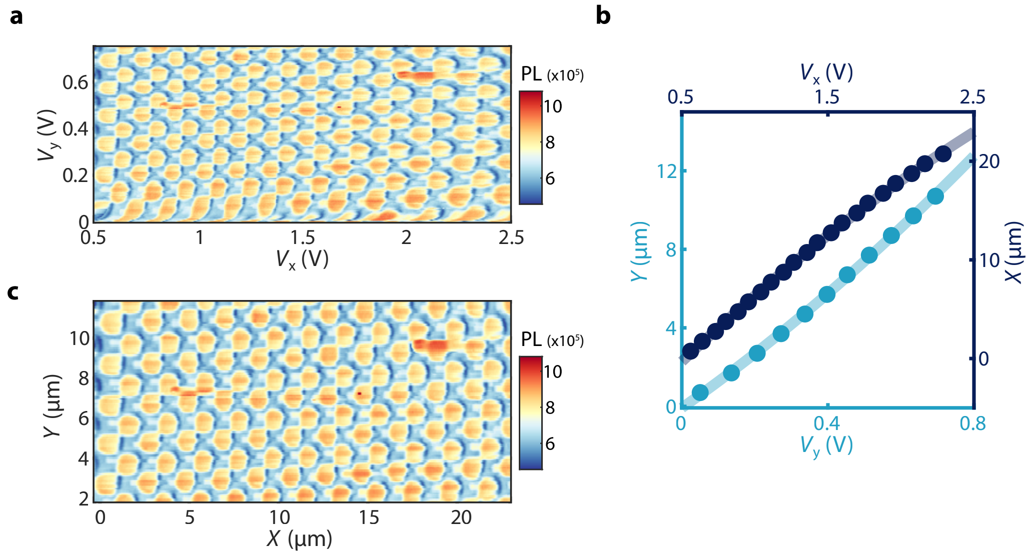


**Figure S.8: Calibration of lateral piezoelectric scanners.** (a) 2D spatial scan of calibration sample with a 2 µm pitch using linearly spaced voltage intervals at a scan speed of $0.1\,\mathrm{V\,s^{-1}}$. (b) We fit the known X and Y-displacement of the chess pits (using a single row or column) as a function of applied X and Y voltage. (c) Interpolated data shown in (a), now using linearly spaced position intervals obtained by the fitting functions in (b) and with the first 25 scan lines removed

### S.10.2 Calibration of the NV-to-sample distance

When retracting the NV-tip from the YIG surface, we observe oscillations in the NV PL (Fig. S.9a). We assume that the oscillations are caused by interference between the YIG-surface-reflected laser light and the laser light internally reflected in the diamond tip (akin to the effect leading to Newton rings). The interference leads to PL oscillations with a spatial period equal to half the laser wavelength (i.e. 515 nm/2). We use these oscillations to estimate the tip-sample distance $d$ for each setpoint distance obtained from the linear voltage-to-distance conversion provided by the supplier of the scanners (Fig. S.9b) .

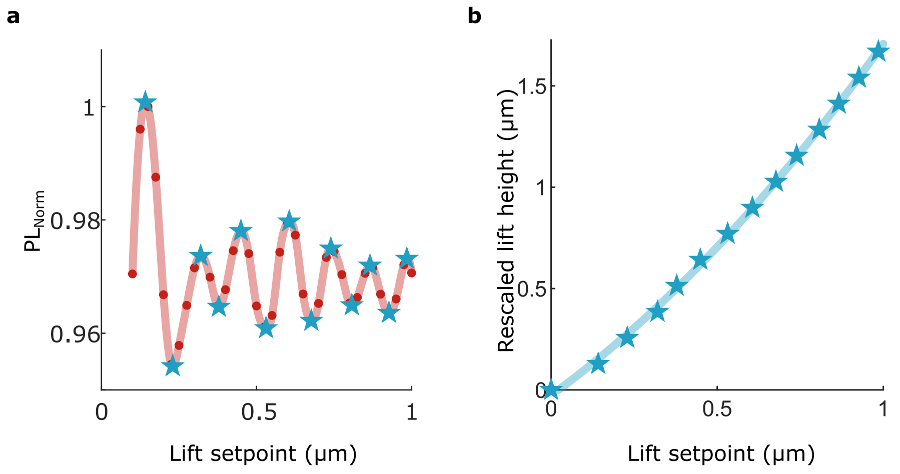


**Figure S.9: Calibration of lift height.** (a) Oscillating PL signal when the tip is retracted from the YIG surface. The stars are the found extrema. Data corresponds to the Rabi measurement shown in Fig. 2b of the main text. (b) We allocate a distance of $\lambda/4$ between each extremum (stars), of which the first maximum corresponds to an absolute distance of $\lambda/4$ from the surface. Our contact position is fixed at zero lift height. We use a second order polynomial to fit the displacement (solid line).

# References


(1) Greentree, A. D.; Olivero, P.; Draganski, M.; Trajkov, E.; Rabeau, J. R.; Reichart, P.; Gibson, B. C.; Rubanov, S.; Huntington, S. T.; Jamieson, D. N.; Prawer, S. Critical components for diamond-based quantum coherent devices. *Journal of Physics: Condensed Matter* **2006**, *18*, S825–S842.

(2) Bertelli, I.; Carmiggelt, J. J.; Yu, T.; Simon, B. G.; Pothoven, C. C.; Bauer, G. E. W.; Blanter, Y. M.; Aarts, J.; Van Der Sar, T. Magnetic resonance imaging of spin-wave transport and interference in a magnetic insulator. *Science Advances* **2020**, *6*, eabd3556.

(3) Zhou, T. X.; Carmiggelt, J. J.; Gächter, L. M.; Esterlis, I.; Sels, D.; Stöhr, R. J.; Du, C.; Fernandez, D.; Rodriguez-Nieva, J. F.; Büttner, F.; Demler, E.; Yacoby, A. A magnon scattering platform. *Proceedings of the National Academy of Sciences* **2021**, *118*, e2019473118.

(4) Rustagi, A.; Bertelli, I.; Van Der Sar, T.; Upadhyaya, P. Sensing chiral magnetic noise via quantum impurity relaxometry. *Physical Review B* **2020**, *102*, 220403(R).

(5) Dréau, A.; Lesik, M.; Rondin, L.; Spinicelli, P.; Arcizet, O.; Roch, J. F.; Jacques, V. Avoiding power broadening in optically detected magnetic resonance of single NV defects for enhanced dc magnetic field sensitivity. *Physical Review B* **2011**, *84*, 195204.

(6) Groß, F.; Zelent, M.; Träger, N.; Förster, J.; Sanli, U. T.; Sauter, R.; Decker, M.; Back, C. H.; Weigand, M.; Keskinbora, K.; Schütz, G.; Krawczyk, M.; Gräfe, J. Building Blocks for Magnon Optics: Emission and Conversion of Short Spin Waves. *ACS Nano* **2020**, *14*, 17184–17193.

(7) Gräfe, J.; Gruszecki, P.; Zelent, M.; Decker, M.; Keskinbora, K.; Noske, M.; Gawronski, P.; Stoll, H.; Weigand, M.; Krawczyk, M.; Back, C. H.; Goering, E. J.; Schütz, G. Direct observation of spin-wave focusing by a Fresnel lens. *Physical Review B* **2020**, *102*, 024420.

(8) Gieniusz, R.; Ulrichs, H.; Bessonov, V. D.; Guzowska, U.; Stognii, A. I.; Maziewski, A. Single antidot as a passive way to create caustic spin-wave beams in yttrium iron garnet films. *Applied Physics Letters* **2013**, *102*, 102409.
